# Case report: Overlap syndrome of neuromyelitis optica spectrum disorder with anti-Argonaute antibodies

**DOI:** 10.3389/fimmu.2024.1366531

**Published:** 2024-06-03

**Authors:** Pei Liu, Xuemei Lin, Songdi Wu

**Affiliations:** ^1^ Department of Neurology, The First Affiliated Hospital of Northwest University, Xi’an, Shaanxi, China; ^2^ Xi’an Key Laboratory for Innovation and Translation of Neuroimmunological Diseases, Xi’an, China

**Keywords:** neuromyelitis optica spectrum disorder, aquaporin-4 antibody, argonaute antibody, Brainstem encephalitis, longitudinally extensive myelitis

## Abstract

Aquaporin-4 antibodies (AQP4-Abs) are a diagnostic marker for patients with a demyelinating disease called neuromyelitis optica spectrum disorder (NMOSD). Anti-Argonaute antibodies (AGO-Abs) present as potential biomarkers of the overlap syndrome between NMOSD and other autoimmune diseases. In this paper, we present the case of an adult woman with numbness, tingling, and burning sensations in her arms and subsequent bilateral internuclear ophthalmoplegia. Brain–cervical–thoracic magnetic resonance imaging (MRI) showed T2 hyperintensities in the dorsal brainstem and around the midbrain aqueduct and longitudinally transverse myelitis with homogeneous enhancement on gadolinium-enhanced MRI. The contemporaneous detection of AQP4- and AGO-Abs led to a definite diagnosis of overlap syndrome of NMOSD with AGO-Abs. The patient was treated with immunosuppressive agents, including corticosteroids and immunoglobulins, and achieved remission. This case highlights a novel phenotype of NMOSD with AGO-Abs overlap syndrome, which presents with relapsing brainstem syndrome and longitudinally extensive myelitis with acute severe neurological involvement. The promising prognosis of the disease could serve as a distinct clinical profile. Broad screening for antibodies against central nervous system autoimmune antigens is recommended in suspected patients with limited or atypical clinical manifestations.

## 1 Introduction

Anti-aquaporin 4 antibodies (AQP4-Abs) specifically target AQP4, a water channel protein found in the central nervous system (CNS) (1, 2). These antibodies are particularly relevant in neuromyelitis optica spectrum disorder (NMOSD), a rare autoimmune disease affecting the optic nerves and spinal cord, and are important diagnostic tools for this disorder (3). The presence of these antibodies in a patient’s blood serum and cerebrospinal fluid (CSF) can enable a definitive diagnosis and differentiate NMOSD from other similar neurological conditions (4, 5).

Argonaute antibodies (AGO-Abs) target proteins involved in gene regulation, specifically in ribonucleic acid (RNA) interference (6). These proteins are involved in the binding and degradation of specific RNA molecules, thereby influencing gene expression (7). AGO-Abs have the potential to identify novel biomarkers for diseases and have been identified in patients with systemic lupus erythematosus, scleroderma, dermatomyositis, and primary Sjögren’s syndrome (pSS), revealing a link with system autoimmunity, sensory neuronopathies, limbic encephalitis, cerebellar syndrome, opsoclonus–myoclonus, length-dependent polyneuropathies (8–11), and NMOSD (12).

We report a case of NMOSD with AGO-Abs, aiming to explore the novel and distinct pattern of NMOSD and expand the clinical spectrum of this rare autoimmune disease.

## 2 Case report

A 63-year-old woman with a history of well-controlled hypertension presented with lumbar tingling and numbness in the right upper limb, chest, and back for 5 months and aggravated binocular diplopia for 1 week. The patient denied having a rash, joint pain, and dry mouth or eyes. The patient had developed episodic chest tingling without obvious causes 5 months prior. She had been diagnosed with shingles at a local clinic. Her symptoms were completely relieved after a local corticosteroid injection. Two months ago, she developed numbness, tingling, and burning sensations in the right arm, chest, and back that had been mistaken as a recurrence of shingles by the local clinic. One week before hospitalization, the patient developed diplopia and was admitted to our hospital with a presumptive diagnosis of myelitis.

Four years previously, the patient was treated for dizziness and binocular diplopia for 2 months. Physical examination revealed bilateral internuclear ophthalmoplegia with slight blepharoptosis and vertical nystagmus. Brain magnetic resonance imaging (MRI) revealed abnormal signals in the tegmentum of the pons and periependymal brainstem lesions (**Supplementary Figure S1**). The serum anti-SSA/Ro-52 antibody test was weakly positive. The serum and CSF of the patient tested negative for CNS demyelinating antibodies, including AQP4-Abs and myelin oligodendrocyte glycoprotein antibodies (MOG-Abs) and oligoclonal bands (OCBs). At the time, brainstem encephalitis was suspected, and methylprednisolone pulse therapy was administered. After 14 days of hospitalization, the condition of the patient significantly improved. When she was discharged, her binocular diplopia improved. No complaints of clinical discomfort were reported when followed up 3 months later.

The patient’s vital signs on admission included a body temperature of 36.2°C, pulse 67 bpm, breathing 18 times/min, blood pressure of 112/83 mmHg, and a body mass index of 31.04 kg/m^2^. Medical examinations revealed abdominal distension. However, the cardiopulmonary and abdominal examinations found no obvious abnormalities. On neurological examination, she was lethargic and unable to follow orders fully. Neuro-ophthalmological examination revealed an oblique right eye, binocular gaze palsy, and vertical nystagmus in both eyes. She exhibited impaired discrimination of the sharp and dull in two trigeminal branches of the right face and mild facial palsy. Muscle weakness of the right lower limb was grade 4 according to the British Medical Research Council. No motor deficits of the upper limbs or left lower limb were detected. Moderate impairment was evident in superficial sensation and tuning fork vibration sensation in the upper limbs, right chest, and back (above level T10). Moderate limb ataxia in the right lower limb and severe truncal ataxia were observed. The tendinous reflexes of the extremities and bilateral pathological signs were unremarkable. An Expanded Disability Status Scale (EDSS) score of 7.5 was obtained based on the clinical manifestations of ophthalmoplegia, moderate trigeminal damage, marked sensation impairment, severe ataxia, and limited walking ability requiring the assistance of a wheelchair.

On August 15, 2023, an auxiliary examination at the hospital measured normal serum, urine, and stool, as well as the erythrocyte sedimentation rate, liver and kidney function, and electrolyte and blood sugar levels. The serum tested weakly positive for anti-SSA/Ro-52 antibodies. The thyroid function of TSH, T3, and T4 was normal, except for the decreased thyroglobulin (0.19 ng/mL) and the increased thyroglobulin antibodies (1,685.6I U/mL). Humoral immunity tests indicated that the levels of immunoglobulins A, G, E, and M and complement were normal. The tuberculosis bacterial immune response, rapid plasma regain levels, and the HIV, hepatitis B, and hepatitis C antibodies were negative.

**Figure 1:**
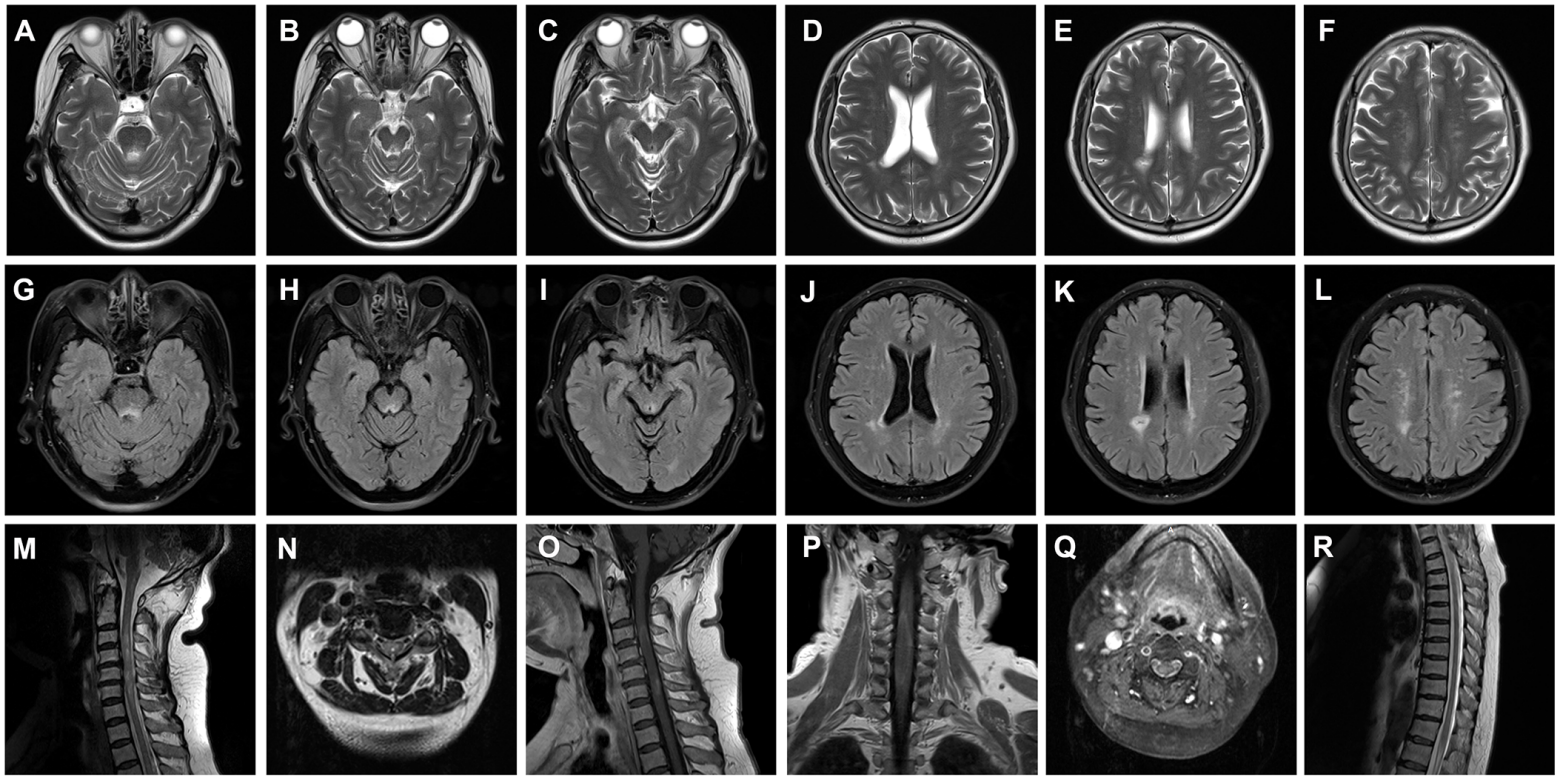
Brain and cervical MRI performed during an acute attack. **(A–L)** Axial T2-weighted **(A–F)** and FLAIR **(G–L)** images of the brain MRI scans showing multifocal patchy or round hyperintense lesions in the superior cerebellar peduncle **(A, G)**, midbrain periaqueduct **(B, C, H, I)**, and left occipital lobe **(C, I)** and deep white matter in the posterior horn of the right lateral ventricle **(D, E, J, K)** and bilateral hemioval center **(F, L)**. Sagittal and axial T2-weighted images of cervical MRI scans show longitudinally transverse lesions along the C3–C7 levels **(M–O)**, with heterogeneous patchy enhancement in the gadolinium (Gd)-enhanced MRI **(P, Q)**. The thoracic spinal cord was normal **(R)**. *MRI*, magnetic resonance imaging; *FLAIR*, fluid-attenuated inversion recovery; *C*, cervical.

**Figure 2:**
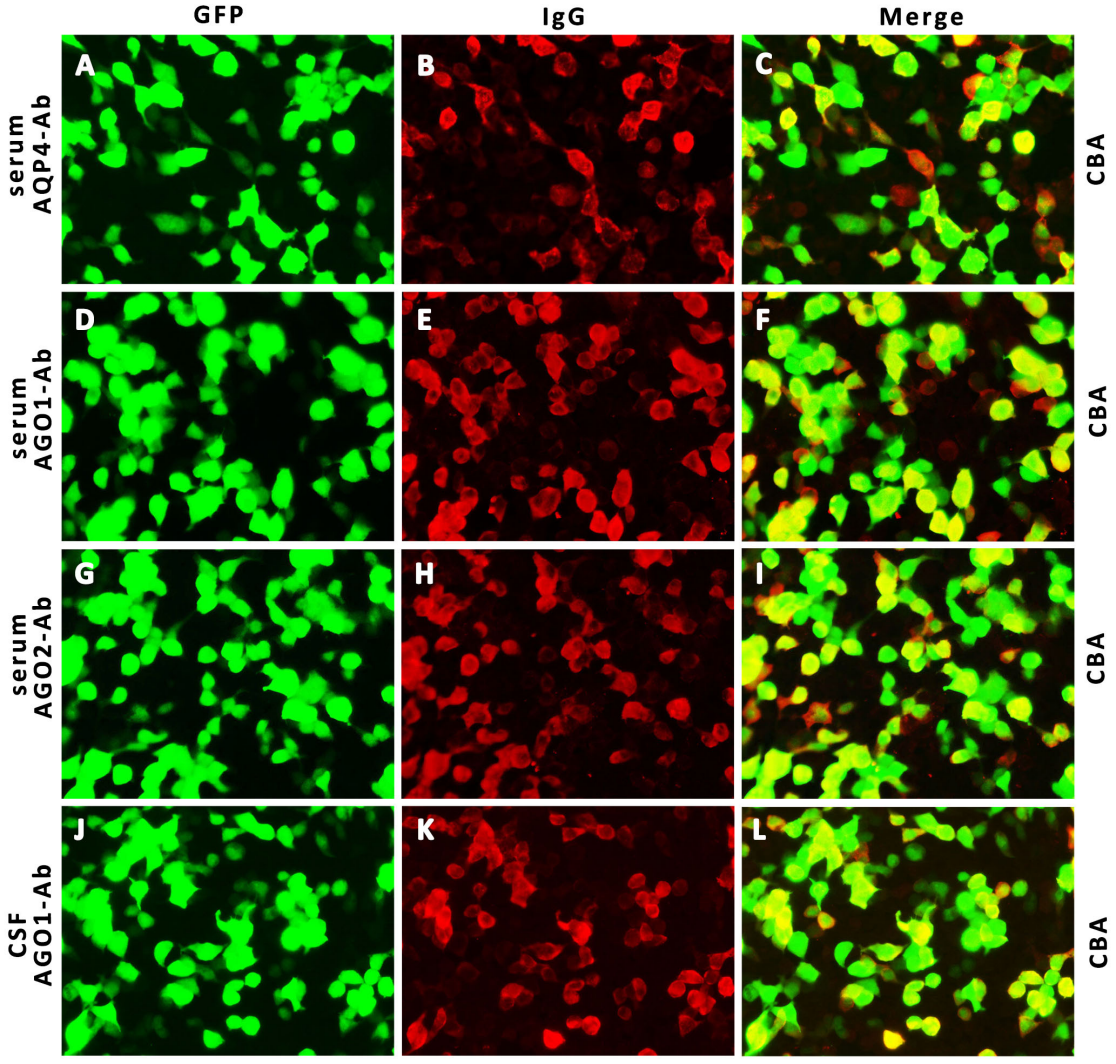
Detection of AQP4- IgG and AGO- IgG by IIF-CBA. Representative CBA photographs show AQP4- IgG in serum (**A–C**, 1:32 titer) and AGO1, 2- IgG in serum (**D–I**, 1:100 titer) and CSF (**J–L**, 1:100 titer). AQP4, neuromyelitis optica spectrum disorder; AGO, argonaute; IIF, indirect immunofluorescence; CBA, cell-based array; CSF, cerebrospinal fluid; GFP, green fluorescent protein; IgG, immunoglobulin G.

**Figure 3:**
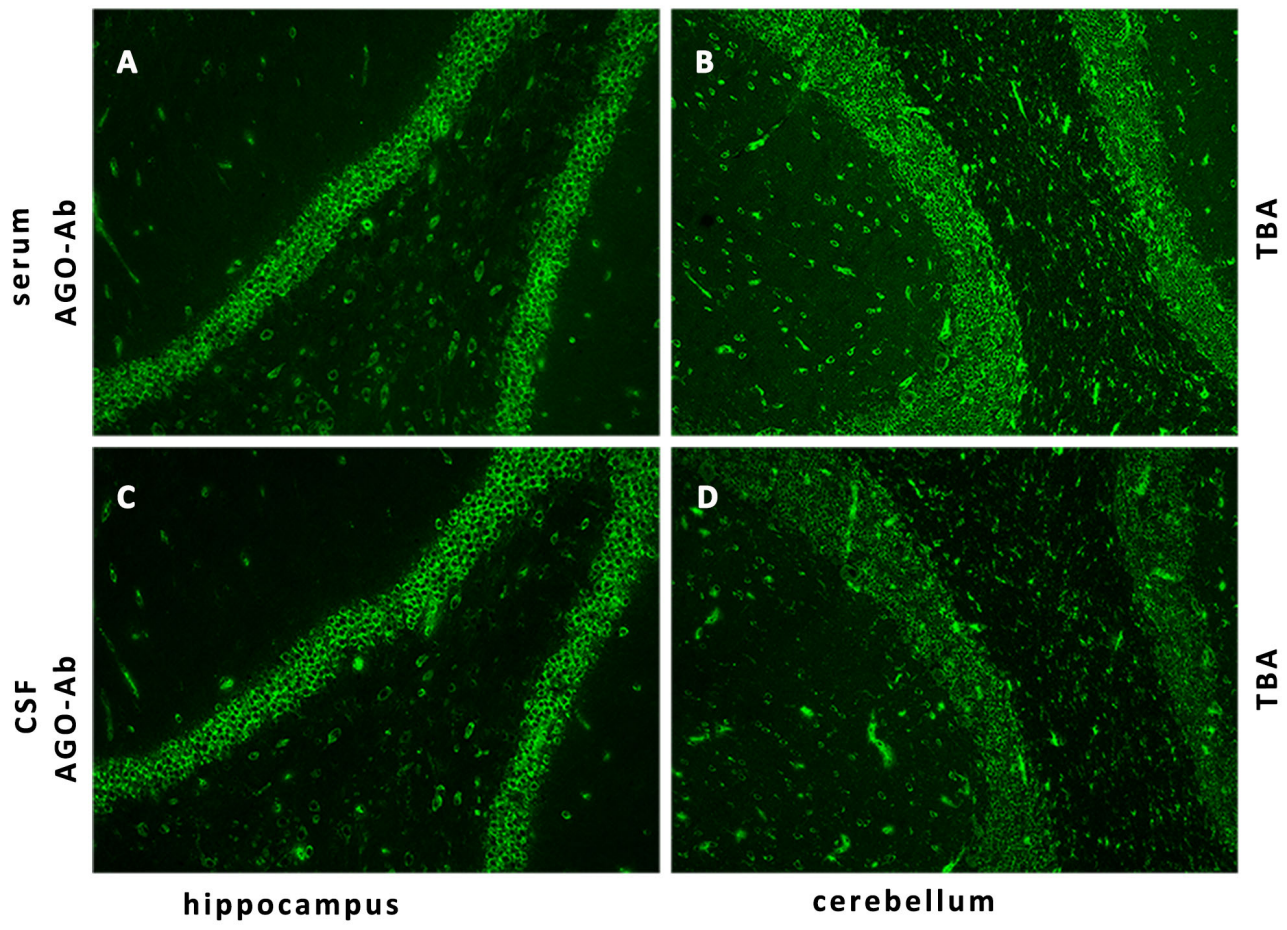
Detection of AGO-IgG by IIF-TBA. IIF of the monkey brain with the patient’s serum **(A, B)** and CSF **(C, D)** shows strong reactivity with the hippocampal stratum pyramidale and cerebellar granule cells. AGO, argonaute; IIF, indirect immunofluorescence; TBA, tissue-based assay.

Her CSF pressure was 210 mmHg. Routine tests revealed normal white blood cells (12 × 10^6^/L), biochemical values (sugar, 4.36 mmol/L; chlorine, 125.1 mmol/L; and protein, 0.23 g/L), and IL-6 (7.84 pg/mL). Acid-quick *Bacillus* ink staining was negative. Axial T2-weighted and fluid-attenuated inversion recovery (FLAIR) images of a brain MRI scan showed multifocal patchy or round hyperintense lesions in the superior cerebellar peduncle, periaqueduct of the midbrain, left occipital lobe, deep white matter in the posterior horn of the right lateral ventricle, and bilateral centrum semiovale (**Figure 1**). The sagittal and axial T2-weighted cervical MRI scans showed longitudinal transverse lesions along the C3–C7 levels, with homogeneous patchy enhancement on gadolinium (Gd)-enhanced MRI (**Figure 1**). The Gd-enhanced MRI showed no enhancement of the brain lesions (**Supplementary Figure S2**). As the autoimmune antibody tests are not all covered in the medical insurance system, we entrusted a third-party detection institution (KingMed Diagnostics Co., Ltd., Xi’an, China) for assistance in the antibody analysis. We initially tested the blood and CSF for CNS demyelinating antibodies using a cell-based assay (CBA), including autoantibodies against AQP4, MOG, GFAP, and OCBs. The results showed AQP4-Abs in the blood with a titer of 1:32 (**Figure 2**); only OCBs were present in the CSF. This finding indicates that immunoglobulin G (IgG) intrathecal synthesis, possibly another autoantibody, was present in the CNS. Therefore, a tissue-based assay (TBA) of the monkey brain with serum and CSF was performed for autoantibody detection, resulting in strong reactivity with the hippocampal stratum pyramidale and cerebellar granule cells (**Figure 3**). The staining pattern was similar to that of the AGO-Abs. Moreover, CBA method with AGO-HEK293-transfected cells confirmed the AGO1-Abs and AGO2-Abs in serum, and AGO1-Abs in CSF, with the titer of 1:100. Further analysis of the AGO1-IgG subclasses documented a subclass of IgG1, but not IgG2, IgG3, or IgG4. Further chest, abdominal, and pelvic computed tomography was performed, excluding the possibility of malignancy.

The patient was diagnosed with overlap syndrome of NMOSD with AGO-Abs and was administered an intravenous pulse therapy regimen of methylprednisolone (1 g/day for 3 days, 500 mg/day for 3 days, 250 mg/day for 3 days, and 120 mg/day for 3 days, followed by tapering oral prednisone tablets at 60 mg/day) based on expert consensus on the treatment of NMOSD in China (13).

While awaiting plasma exchange, her condition suddenly worsened on the morning of the third day of hospitalization, manifesting as lethargy, chest tightness, dyspnea, and increased weakness in the right lower limb. Urgent blood gas examination indicated an oxygen pressure of 60 mmHg and a carbon dioxide pressure of 49 mmHg. The patient was immediately transferred to the intensive care unit. Her family members were informed that the condition was serious, possibly due to the increase in lesions involving the brainstem heartbeat, respiratory center, and ascending reticular activating system.

After agreement with her husband, the patient’s vital signs were closely monitored and a combination of immunoglobulin treatments was prescribed. After 3 days of rescue treatment, her condition gradually improved, her consciousness became clear, the burning pain in both upper limbs significantly improved, and the muscle strength of the right lower limb gradually overturned.

**Figure 4:**
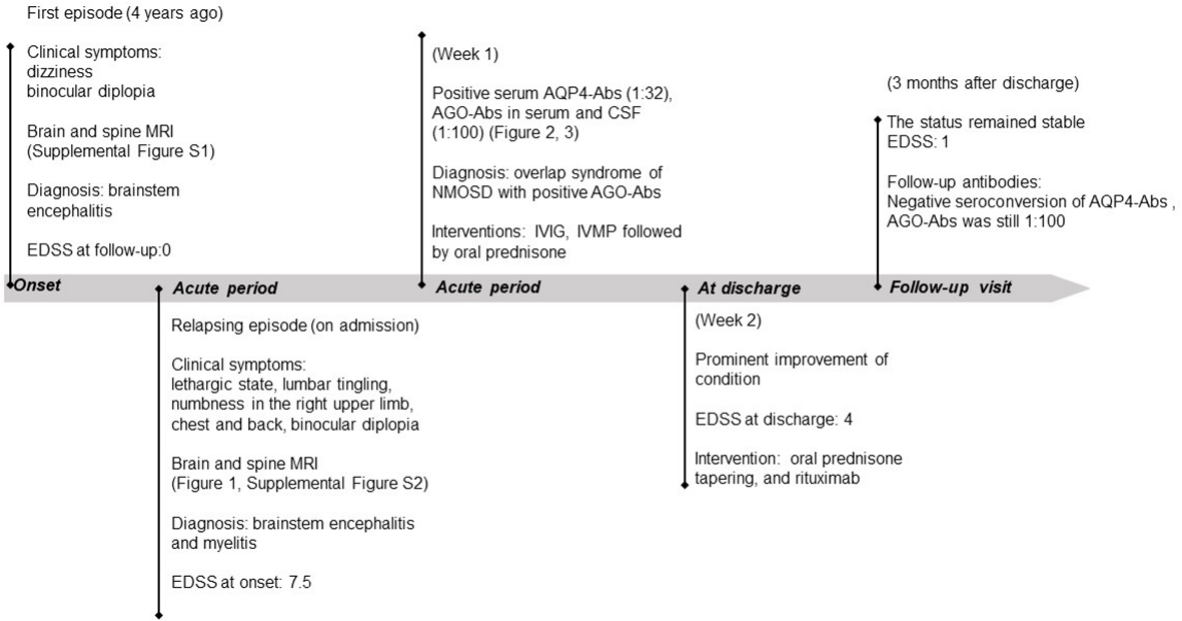
Timeline of the patient, with relevant episode and intervention data. *IVIG*, intravenous immunoglobulin; *IVMP*, intravenous methylprednisolone; *EDSS*, expanded disability status scale.

The EDSS score at discharge was 4.0. We administered a regimen of rituximab (200 mg) for successive weeks as the initial dose to prevent disease recurrence. Her condition improved significantly at the return visit 2 weeks prior, with the EDSS score decreasing to 3.0. During the follow-up visit 3 months after onset, the serum AQP4-Abs and AGO-Abs were re-tested using the CBA. The results showed reverse negativity for serum AQP4-Abs, and the serum remained positive for AGO-Abs with a titer of 1:100. The patient reported no symptoms of discomfort, including joint and muscle pain, after the application of rituximab. She is currently undergoing regular follow-up examinations. The patient’s timeline with relevant data on episodes and interventions is presented in **Figure 4**.

## 3 Discussion

It is well known that the detection of AQP4-Abs in the serum or CSF is a highly specific pathogenic biomarker for the definite diagnosis of NMOSD, combined with characteristic manifestations and neuroimaging. Most individuals with NMOSD have serum antibodies that target AQP4 (3). However, a subgroup of patients test negative for AQP4-Abs, especially those with asymptomatic manifestations or a temporal course with only a single clinical attack, making the diagnosis difficult (14–16). Similarly, although the patient in this study presented with acute brainstem syndrome at the first clinical attack with negative AQP4-Abs in the serum or CSF, the diagnosis was pending until a relapse of brainstem encephalitis with AQP4-Abs in the serum.

Patients with negative AQP4-Abs onset and clinical manifestations that do not meet the diagnostic criteria, including isolated area postrema syndrome (APS), isolated optic neuritis, isolated longitudinally extensive transverse myelitis, simultaneous short-segment transverse myelitis, and other core clinical manifestations, are diagnosed with the inaugural limited form of NMOSD (LF-NMOSD), which precedes future definite NMOSD (15, 17). A prospective cohort study found that the mean interval conversion from AQP4-Abs seronegativity to seropositivity was >36 months (16). In our patient, the time interval from the onset of the first episode to antibody conversion was 4 years. Therefore, we believe that the brainstem syndrome corresponding to the periependymal brainstem lesions would be LF-NMOSD with a high risk of developing definite NMOSD, and long-term follow-up is necessary to trace the propensity for relapse several years later (18). In addition, there is insufficient evidence of relapsing encephalitis within the same brainstem area in patients with NMOSD.

Although the patient was seropositive for anti-SSA antibodies, other characteristic manifestations suggestive of Sjögren’s syndrome were absent, and the diagnostic criteria for pSS were not fulfilled. It was found that in 20 out of 44 (45.5%) cases with overlap syndrome of NMOSD and pSS, the initial neurological event of NMOSD occurred 3.5 years before the onset of pSS (19); however, mild sicca syndrome may not sufficiently fulfill the diagnostic criteria for pSS (19–22), suggesting that anti-SSA/Ro antibodies could be an early predictor of NMOSD in the autoimmune milieu. A retrospective study in China found that approximately 7% (43/616) of eligible patients with pSS developed NMOSD; 51% (22/43) of these patients developed LF-NMOSD. These patients had unique clinical features, including severe neurological deficits and mild or quiescent pSS (22, 23). In the neuroimaging results, CNS lesions are commonly distributed around the periependymal area, brainstem lesions, and cervical and thoracic lesions. The most specific antibodies for pSS are anti-Ro-52 or SSA antibodies (20, 21). These findings are consistent with the clinical features of the patient in this study. They also highlight a potential link between pSS and NMOSD in summary statistical data from genome-wide association studies (24). Further research is needed to fully understand the underlying mechanisms and clinical implications.

This study demonstrated the coalesce of AGO-Abs in NMOSD. AGO-Abs in patients with NMOSD could aid in the diagnosis, monitoring, and prediction of disease activity (12). A prospective study found that AGO-Abs were detected in 6.7% of patients with NMOSD, concluding that AGO-Abs could serve as a biomarker with high specificity and sensitivity for NMOSD (12). Notably, Moritz et al. demonstrated that the ELISA method using an antigen-stabilizing protocol is more sensitive compared with CBA, but with the same specificity (25). Thus, there could be more cases with AGO-Abs in NMOSD than currently reported.

The pathophysiological role of AGO-Abs in patients with NMOSD presenting with myelitis remains unclear. AGO-Abs indicate a more severe clinical manifestation onset with a distinct phenotype of exclusively extensive transverse myelitis and prominent neurological impairment sequelae in NMOSD, sensory neuronopathies, several limbic encephalitis, and cerebellar syndrome (8, 11, 12). AGO proteins are cytoplasmic antigens, and the production of AGO-Abs immediately leads to a severe autoimmune inflammatory reaction (26, 27). In serum samples positive for AGO-Abs, the CBA reaction of AGO1- and AGO2-Abs is more reactive than that of AGO3- and AGO4-Abs. AGO1- and AGO2-Abs mediate more serious autoimmune diseases (8). The blood and CSF samples from our patient were positive for AGO-Abs in the relapse, consistent with a severe condition in the acute stage, suggesting that AGO-Abs exacerbate the immune response of NMOSD, leading to the deterioration of the disease. Among seven patients, only one experienced relapse, which initially presented with diencephalic syndrome and later relapsed with myelitis and APS (12).

Similarly, our patient presented with typical extensive myelitis and recurrent brainstem encephalitis, not exclusive myelitis, suggesting that the clinical characteristics of the overlap syndrome of AGO-IgG in NMOSD tend to be myelitis with APS or brainstem encephalitis during the relapse course. In addition, although the patient had respiratory failure and disturbance of consciousness at the time of onset, her EDSS score improved. Future large cohort studies are warranted to investigate the phenotypic diversity and to clarify whether AGO-Abs are exclusive diagnostic biomarkers for extensive transverse myelitis or brainstem encephalitis in NMOSD.

AGO-Abs are associated explicitly with distinctive clinical characteristics in 5%–20% of patients with systemic rheumatic diseases (8, 28, 29). The hypothesis speculated that Ro proteins are also cytoplasmic proteins involved in the metabolic and posttranscriptional regulation of gene expression or even in patients with peripheral neuropathy as Hu and Ri antigens are RNA binding proteins as well (11, 30). Therefore, the coexistent antibodies can appear simultaneously in specific autoimmune diseases. Similarly, the overlap of AGO-, AQP4-, and Ro-52-Abs in this patient suggests multiple autoimmune conditions or overlapping syndromes. However, there is no available evidence explaining the potential or specific effects of AGO-Abs on the pathogenesis of NMOSD and pSS. The co-association could be caused by the shared genetic and environmental factors predisposing individuals to autoimmunity.

The pathogenesis of the co-occurrence of AQP4-, AGO-, and SSA-Abs in individuals is complex and remains unknown. One possible explanation is epitope spreading, which is characterized by various chronic immune-mediated diseases and contributes to the perpetuation and chronicity of autoimmune responses (31, 32). AGO and Ro are cytoplasmic proteins involved in the metabolic and posttranscriptional regulation of gene expression. However, in our case, it remains unclear whether the AQP4- and AGO-Abs emerged simultaneously or successively. The pathological role and peptide specificity of these antigens could catalyze overlap syndrome. Genetic and environmental factors could also play a role in the coexistence of multiple autoantibodies. Some individuals may have genetic and environmental predispositions that make them more susceptible to autoantibody production from multiple epitopes or autoantigens (33).

Treatment of overlapping syndromes requires a comprehensive and integrated approach that considers several challenges, including the therapeutic options, duration of treatment, and the initiation time of immunosuppressants. Treatment usually involves a multidisciplinary approach (20) that addresses the intricate manifestations of a multi-autoimmune entity. Personalized treatment regimens for patients with NMOSD presenting with AGO-Abs have not been fully studied. The treatment strategies for the overlap syndrome of NMOSD have always been based on extrapolation from studies on NMOSD (8, 12). The first-line therapy in the acute phase is intravenous methylprednisolone, intravenous immunoglobulin (IVIG), or plasma exchange, followed by oral corticosteroid tapering, the most prevalent first-line treatment during acute attacks. Sensory neuronopathy combined with AGO1-Abs has also been reported to have a positive effect on IVIG therapy (11). The condition of the patient in this study deteriorated during treatment with a methylprednisolone pulse. However, it improved significantly 3 days after combining it with IVIG, suggesting that the response to immunomodulatory treatment is associated with AGO-Abs. Patients are also recommended to receive treatment with immunosuppressants or disease-modifying agents during the maintenance period due to the high risk of relapse and long-term severe disability outcomes (18, 34, 35).

## 4 Summary

This case highlights a novel phenotype of the overlap syndrome of NMOSD with AGO-Abs that has not been previously reported. The patient presented with recurrent brainstem encephalitis and longitudinally extensive myelitis, expanding the clinical spectrum of overlap syndrome. Broad screening for antibodies against CNS and systemic autoimmune antigens is recommended in suspected patients with limited or atypical clinical manifestations. Active immunosuppressants are recommended to prevent relapse and disability progression. Considering the low proportion of patients who are anti-AGO antibody-positive in clinical practice, clinicians should pay close attention to this rare overlap syndrome. Further research is necessary to elucidate the underlying mechanisms and to establish optimal management strategies for patients with this rare combination of autoimmune conditions.

## Data availability statement

The original contributions presented in the study are included in the article/**Supplementary Material**. Further inquiries can be directed to the corresponding author.

## Ethics statement

The studies involving human participant was reviewed and approved by the Ethics Committees of The First Affiliated Hospital of Northwest University. The patient provided her written informed consent to participate in this study. Written informed consent was obtained from the participant/patient(s) for the publication of this case report.

## Author contributions

PL: Data curation, Formal analysis, Investigation, Supervision, Writing – original draft, Writing – review & editing. XL: Conceptualization, Investigation, Supervision, Writing – review & editing. SW: Conceptualization, Funding acquisition, Investigation, Validation, Writing – review & editing.

## Supplementary material

Supplementary Figure 1 Brain and cervical magnetic resonance imaging (MRI) performed during the first episode of an acute attack. **(A, B)** Axial T2-weighted brain MRI shows hyperintense lesions around the midbrain periaqueduct. **(C)** Sagittal fluid-attenuated inversion recovery (FLAIR) images show diffuse and extensive demyelinating lesions throughout the midbrain. **(D)** Sagittal T2-weighted cervical MRI scans are unremarkable.

Supplementary Figure 2 Brain gadolinium (Gd)-enhanced MRI is normal without enhancement of brain lesions during the relapse of an acute attack.

## References

[1] Chihara N Yamamura T . Immuno-pathogenesis of neuromyelitis optica and emerging therapies. Semin immunopathol. (2022) 44:599–610. doi: 10.1007/s00281-022-00941-9 35635574

[2] Wingerchuk DM Banwell B Bennett JL Cabre P Carroll W Chitnis T . International consensus diagnostic criteria for neuromyelitis optica spectrum disorders. Neurology. (2015) 85:177–89. doi: 10.1212/WNL.0000000000001729 PMC4515040 26092914

[3] Jarius S Aktas O Ayzenberg I Bellmann-Strobl J Berthele A Giglhuber K . Update on the diagnosis and treatment of neuromyelits optica spectrum disorders (NMOSD) - revised recommendations of the Neuromyelitis Optica Study Group (NEMOS). Part I: Diagnosis and differential diagnosis. J Neurol. (2023) 270:3341–68. doi: 10.1007/s00415-023-11634-0 PMC10267280 37022481

[4] Jarius S Wildemann B . The history of neuromyelitis optica. J Neuroinflamm. (2013) 10:8. doi: 10.1186/1742-2094-10-8 PMC3599417 23320783

[5] Jarius S Wildemann B . The history of neuromyelitis optica. Part 2: ‘Spinal amaurosis’, or how it all began. J Neuroinflamm. (2019) 16:280. doi: 10.1186/s12974-019-1594-1 PMC6935230 31883522

[6] Jakymiw A Ikeda K Fritzler MJ Reeves WH Satoh M Chan EK . Autoimmune targeting of key components of RNA interference. Arthritis Res Ther. (2006) 8:R87. doi: 10.1186/ar1959 16684366 PMC1779426

[7] Pruijn GJ . The RNA interference pathway: a new target for autoimmunity. Arthritis Res Ther. (2006) 8:110. doi: 10.1186/ar1987 16805905 PMC1779402

[8] Do LD Moritz CP Muniz-Castrillo S Pinto AL Tholance Y Brugiere S . Argonaute autoantibodies as biomarkers in autoimmune neurologic diseases. Neurol Neuroimmunol Neuroinflamm. (2021) 8:e1032. doi: 10.1212/NXI.0000000000001032 34321331 PMC8362341

[9] Satoh M Langdon JJ Chou CH McCauliffe DP Treadwell EL Ogasawara T . Characterization of the Su antigen, a macromolecular complex of 100/102 and 200-kDa proteins recognized by autoantibodies in systemic rheumatic diseases. Clin Immunol immunopathol. (1994) 73:132–41. doi: 10.1006/clin.1994.1179 7923910

[10] Treadwell EL Alspaugh MA Sharp GC . Characterization of a new antigen-antibody system (Su) in patients with systemic lupus erythematosus. Arthritis rheuma. (1984) 27:1263–71. doi: 10.1002/art.1780271108 6497921

[11] Moritz CP Tholance Y Vallayer PB Do LD Muniz-Castrillo S Rogemond V . Anti-AGO1 antibodies identify a subset of autoimmune sensory neuronopathy. Neurol Neuroimmunol Neuroinflamm. (2023) 10:e200105. doi: 10.1212/NXI.0000000000200105 37072227 PMC10112859

[12] Carta S Le Duy D Rogemond V Derache N Chaumont H Fromont A . Anti-Argonaute antibodies as a potential biomarker in NMOSD. J neurol neurosurg Psychiatry. (2023) 94:738–41. doi: 10.1136/jnnp-2022-330707 36810322

[13] Chinese Society of Immunology Neuroimmunology Branch . Guidelines for diagnosis and treatment of neuromyelitis spectrum diseases in China (2021). Chin J Neuroimmunol Neurol. (2021) 28:423–36. doi: 10.3969/j.issn.1006-2963.2021.06.002

[14] Wu Y Geraldes R Jurynczyk M Palace J . Double-negative neuromyelitis optica spectrum disorder. Mult Scler. (2023) 29:1353–1362. doi: 10.1177/13524585231199819 PMC10580671 37740717

[15] Sepulveda M Armangue T Sola-Valls N Arrambide G Meca-Lallana JE Oreja-Guevara C . Neuromyelitis optica spectrum disorders: Comparison according to the phenotype and serostatus. Neurol Neuroimmunol Neuroinflamm. (2016) 3:e225. doi: 10.1212/NXI.0000000000000225 27144216 PMC4841645

[16] Chen X Zhou J Li R Zhang B Wang Y Zhong X . Disease course and outcomes in patients with thelimited form of neuromyelitis optica spectrum disorders and negative AQP4-IgG serology at disease onset: a prospective cohort study. J Clin Neurol. (2022) 18:453–62. doi: 10.3988/jcn.2022.18.4.453 PMC9262456 35796271

[17] Sato DK Lana-Peixoto MA Fujihara K de Seze J . Clinical spectrum and treatment of neuromyelitis optica spectrum disorders: evolution and current status. Brain Pathol. (2013) 23:647–60. doi: 10.1111/bpa.12087 PMC8029254 24118482

[18] Kumpfel T Giglhuber K Aktas O Ayzenberg I Bellmann-Strobl J Haussler V . Update on the diagnosis and treatment of neuromyelitis optica spectrum disorders (NMOSD) - revised recommendations of the Neuromyelitis Optica Study Group (NEMOS). Part II: Attack therapy and long-term management. J Neurol. (2023) 271:141–76. doi: 10.1007/s00415-023-11910-z PMC10770020 37676297

[19] Prasad CB Kopp CR Naidu G Sharma V Misra DP Agarwal V . Overlap syndrome of anti-aquaporin 4 positive neuromyelitis optica spectrum disorder and primary Sjogren’s syndrome: a systematic review of individual patient data. Rheumatol Int. (2023). doi: 10.1007/s00296-023-05397-0 37500817

[20] Qiao L Wang Q Fei Y Zhang W Xu Y Zhang Y . The clinical characteristics of primary Sjogren’s syndrome with neuromyelitis optica spectrum disorder in China: a STROBE-compliant article. Medicine. (2015) 94:e1145. doi: 10.1097/MD.0000000000001145 26181553 PMC4617097

[21] Kahlenberg JM . Neuromyelitis optica spectrum disorder as an initial presentation of primary Sjogren’s syndrome. Semin Arthritis rheuma. (2011) 40:343–8. doi: 10.1016/j.semarthrit.2010.05.005 20655576

[22] Min JH Kim HJ Kim BJ Lee KW Sunwoo IN Kim SM . Brain abnormalities in Sjogren syndrome with recurrent CNS manifestations: association with neuromyelitis optica. Mult Scler. (2009) 15:1069–76. doi: 10.1177/1352458509106228 19625331

[23] Martin-Nares E Hernandez-Molina G Fragoso-Loyo H . Aquaporin-4-IgG positive neuromyelitis optica spectrum disorder and systemic autoimmune diseases overlap syndrome: a single-center experience. Lupus. (2019) 28:1302–11. doi: 10.1177/0961203319877255 31566079

[24] Wang X Shi Z Zhao Z Chen H Lang Y Kong L . The causal relationship between neuromyelitis optica spectrum disorder and other autoimmune diseases. Front Immunol. (2022) 13:959469. doi: 10.3389/fimmu.2022.959469 36248893 PMC9562912

[25] Moritz CP Do LD Tholance Y Vallayer PB Rogemond V Joubert B . Conformation-stabilizing ELISA and cell-based assays reveal patient subgroups targeting three different epitopes of AGO1 antibodies. Front Immunol. (2022) 13:972161. doi: 10.3389/fimmu.2022.972161 36341350 PMC9630334

[26] Peters L Meister G . Argonaute proteins: mediators of RNA silencing. Mol Cell. (2007) 26:611–23. doi: 10.1016/j.molcel.2007.05.001 17560368

[27] Meister G Landthaler M Peters L Chen PY Urlaub H Luhrmann R . Identification of novel argonaute-associated proteins. Curr biol: CB. (2005) 15:2149–55. doi: 10.1016/j.cub.2005.10.048 16289642

[28] Filkova M Jungel A Gay RE Gay S . MicroRNAs in rheumatoid arthritis: potential role in diagnosis and therapy. BioDrugs: Clin immunother biopharmaceutic Gene Ther. (2012) 26:131–41. doi: 10.2165/11631480-000000000-00000 22494429

[29] Ogawa-Momohara M Muro Y Satoh M Akiyama M . Autoantibodies to Su/Argonaute 2 in Japanese patients with inflammatory myopathy. Clinica chimica acta; Int J Clin Chem. (2017) 471:304–7. doi: 10.1016/j.cca.2017.06.022 28673815

[30] Ceribelli A Tincani A Cavazzana I Franceschini F Cattaneo R Pauley BA . Anti-argonaute2 (Ago2/Su) and -Ro antibodies identified by immunoprecipitation in primary anti-phospholipid syndrome (PAPS). Autoimmunity. (2011) 44:90–7. doi: 10.3109/08916934.2010.499886 20695766

[31] Sinmaz N Nguyen T Tea F Dale RC Brilot F . Mapping autoantigen epitopes: molecular insights into autoantibody-associated disorders of the nervous system. J Neuroinflamm. (2016) 13:219. doi: 10.1186/s12974-016-0678-4 PMC5006540 27577085

[32] Vanderlugt CL Miller SD . Epitope spreading in immune-mediated diseases: implications for immunotherapy. Nat Rev Immunol. (2002) 2:85–95. doi: 10.1038/nri724 11910899

[33] Hor JY Asgari N Nakashima I Broadley SA Leite MI Kissani N . Epidemiology of neuromyelitis optica spectrum disorder and its prevalence and incidence worldwide. Front Neurol. (2020) 11:501. doi: 10.3389/fneur.2020.00501 32670177 PMC7332882

[34] Lin L Hang H Zhang J Lu J Chen D Shi J . Clinical significance of anti-SSA/Ro antibody in Neuromyelitis optica spectrum disorders. Mult Scler Relat Disord. (2022) 58:103494. doi: 10.1016/j.msard.2022.103494 35051897

[35] Cruz RA Chaudhary S Guevara M Meltzer E . Neuromyelitis optica spectrum disorders (NMOSD) and connective tissue disease (CTD): an update for the rheumatologist. Curr Rheumatol Rep. (2021) 23:33. doi: 10.1007/s11926-021-01000-2 33909180

