# Supplementary figures and images for: Case report: Overlap syndrome of neuromyelitis optica spectrum disorder with anti-Argonaute antibodies
Source: Front Immunol. 2024 Jun 3;15:1366531. doi: 10.3389/fimmu.2024.1366531 (PMC11180789; doi:10.3389/fimmu.2024.1366531)

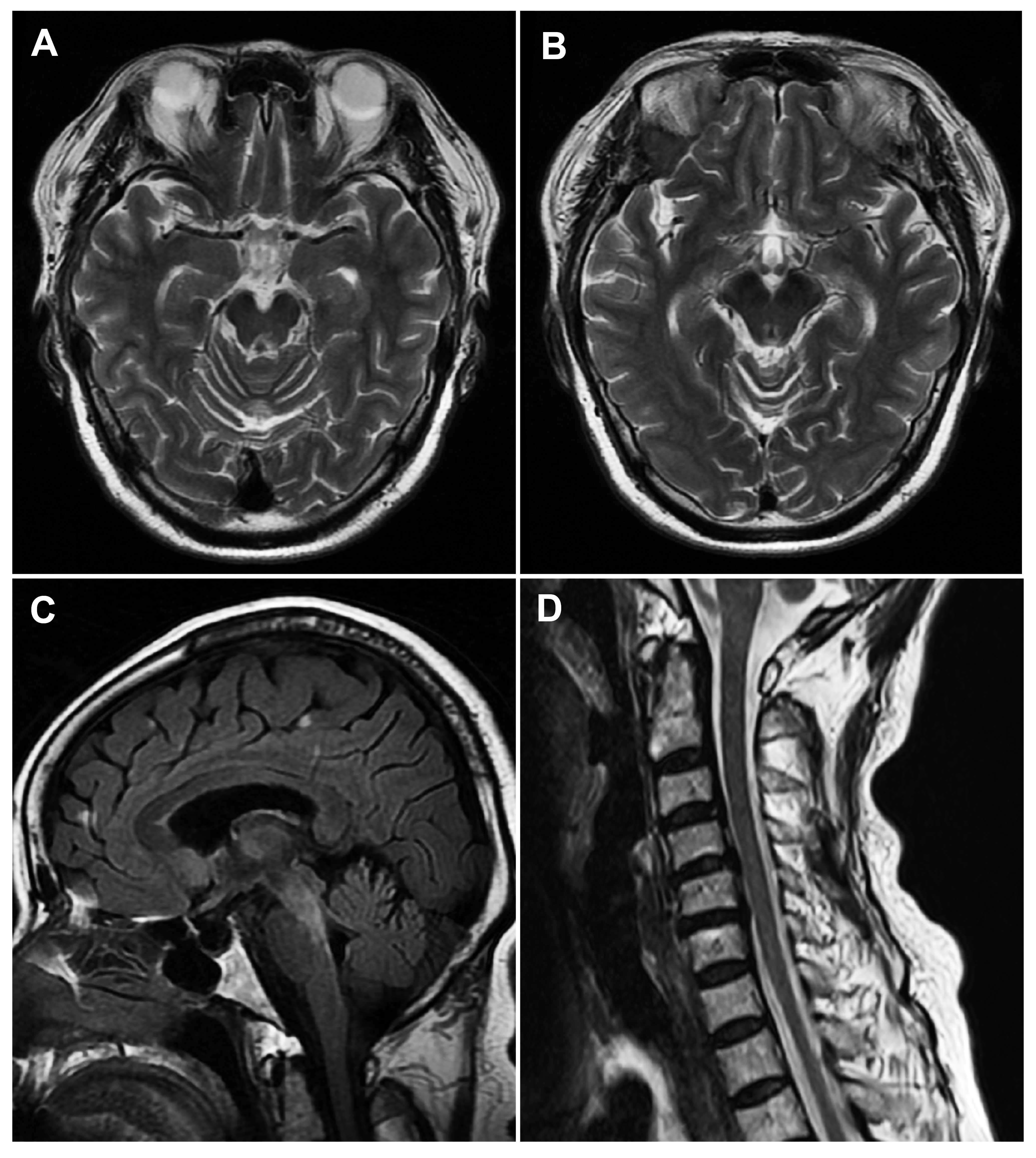

Supplement: Supplementary Figure 1 — Brain and cervical magnetic resonance imaging (MRI) performed during the first episode of an acute attack. (A, B) Axial T2-weighted brain MRI shows hyperintense lesions around the midbrain periaqueduct. (C) Sagittal fluid-attenuated inversion recovery (FLAIR) images show diffuse and extensive demyelinating lesions throughout the midbrain. (D) Sagittal T2-weighted cervical MRI scans are unremarkable. [file Image_1.jpeg]

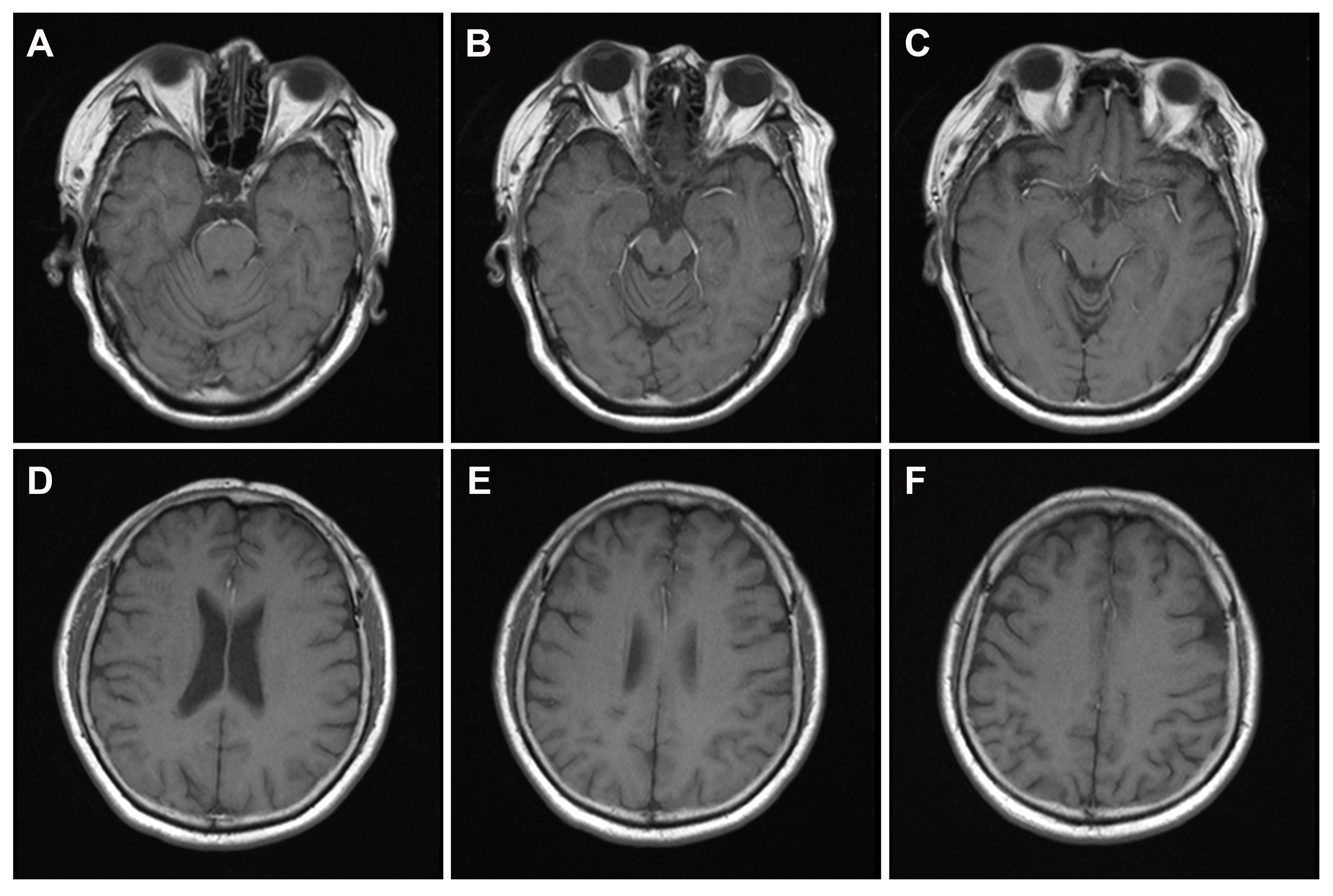

Supplement: Supplementary Figure 2 — Brain gadolinium (Gd)-enhanced MRI is normal without enhancement of brain lesions during the relapse of an acute attack. [file Image_2.jpeg]
